# Modulation of K_V_7 Channel Deactivation by PI(4,5)P_2_

**DOI:** 10.3389/fphar.2020.00895

**Published:** 2020-06-19

**Authors:** Carlos A. Villalba-Galea

**Affiliations:** Department of Physiology and Pharmacology, Thomas J. Long School of Pharmacy, University of the Pacific, Stockton, CA, United States

**Keywords:** K_V_7 channels, phosphoinositide, voltage-sensitive phosphatases, Retigabine, PI(4,5)P_2_, hysteresis

## Abstract

The activity of K_V_7 channels critically contributes to the regulation of cellular electrical excitability in many cell types. In the central nervous system, the heteromeric K_V_7.2/K_V_7.3 channel is thought to be the chief molecular entity giving rise to M-currents. These K^+^-currents as so called because they are inhibited by the activation of Gq protein-coupled muscarinic receptors. In general, activation of Gq protein-coupled receptors (GqPCRs) decreases the concentration of the phosphoinositide PI(4,5)P_2_ which is required for K_V_7 channel activity. It has been recently reported that the deactivation rate of K_V_7.2/K_V_7.3 channels decreases as a function of activation. This suggests that the activated/open channel stabilizes as activation persists. This property has been regarded as evidence for the existence of modal behavior in the activity of these channels. In particular, it has been proposed that the heteromeric K_V_7.2/K_V_7.3 channel has at least two modes of activity that can be distinguished by both their deactivation kinetics and sensitivity to Retigabine. The current study was aimed at understanding the effect of PI(4,5)P_2_ depletion on the modal behavior of K_V_7.2/K_V_7.3 channels. Here, it was hypothesized that depleting the membrane of P(4,5)P_2_ would hamper the stabilization of the activated/open channel, resulting in higher rates of deactivation of the heteromeric K_V_7.2/K_V_7.3 channel. In addressing this question, it was found that the activity-dependent slowdown of the deactivation was not as prominent when channels were co-expressed with the chimeric phosphoinositide-phosphatase Ci-VS-TPIP or when cells were treated with the phosphoinositide kinase inhibitor Wortmannin. Further, it was observed that either of these approaches to deplete PI(4,5)P_2_ had a higher impact on the kinetic of deactivation following prolonged activation, while having little or no effect when activation was short-lived. Furthermore, it was observed that the action of either Ci-VS-TPIP or Wortmannin reduced the effect of Retigabine on the kinetics of deactivation, having a higher impact when activation was prolonged. These combined observations led to the conclusion that the deactivation kinetic of K_V_7.2/K_V_7.3 channels was sensitive to PI(4,5)P_2_ depletion in an activation-dependent manner, displaying a stronger effect on deactivation following prolonged activation.

## Introduction

To the memory of Louis J. De Felice. A great scientist, an inspiring mentor, and, simply, a great friend. “…never forget the physics behind it!”

Voltage-gated, potassium-selective (K_V_) channels from the K_V_7 family are commonly found in the cardiovascular, gastrointestinal, and nervous systems. They are the main molecular entities responsible for the so-called M-currents which are voltage-dependent K^+^-currents suppressed by the activity of Gq protein-coupled receptors (GqPCRs) (Brown and Adams, 1980; Wang et al., 1998; Jentsch, 2000; Cooper, 2012). K_V_7 channels can display measurable activity at voltages as negative as −60 mV, making them into critical contributors to the maintenance of the resting membrane potential (Brown and Adams, 1980; Wang et al., 1998; Jentsch, 2000; Cooper, 2012; Linley et al., 2012; Soh et al., 2014; Mastrangelo, 2015; Miceli et al., 2015). In the nervous system, K_V_7 channels are critically involved in controlling excitability in neurons. Indeed, mutations that impair the normal functioning of K_V_7 channels cause neurological disorders such as Benign Familial Neonatal Seizures (Charlier et al., 1998; Singh et al., 1998; Dedek et al., 2001; Wuttke et al., 2008), Early Onset Epileptic Encephalopathy (Weckhuysen et al., 2012; Weckhuysen et al., 2013; Mastrangelo, 2015; Miceli et al., 2015; Abidi et al., 2015), and Peripheral Nerve Hyperexcitability (Dedek et al., 2001; Wuttke et al., 2007).

The role of K_V_7 channels in the control of excitability in electrically active cells have been long recognized. So has been the potential of these proteins as therapeutic targets for hyperexcitability disorders such as chronic pain, epilepsy, and cardiac arrhythmias (Brown and Adams, 1980; Wang et al., 1998; Jentsch, 2000; Cooper, 2012; Linley et al., 2012; Soh et al., 2014; Mastrangelo, 2015; Miceli et al., 2015). For instance, Retigabine, a first-in-class anticonvulsant, was recently used in the U.S. to treat epileptic disorders in adults (Rundfeldt and Netzer, 2000; Wickenden et al., 2000). This drug is a K_V_7 agonist that facilitates the opening of all members of the K_V_7 family, except for K_V_7.1 (Main et al., 2000; Schenzer et al., 2005; Wuttke et al., 2005). In the presence of Retigabine, the voltage-dependence for activation of K_V_7.2 and K_V_7.3 channels shifts toward more negative potentials, reaching up to a −40-mV shift at a concentration of 100 µM of this drug (Rundfeldt and Netzer, 2000; Wickenden et al., 2000; Main et al., 2000; Rundfeldt and Netzer, 2000; Tatulian and Brown, 2003; Schenzer et al., 2005; Wuttke et al., 2005; Gunthorpe et al., 2012; Kim et al., 2015). This effect made Retigabine into a promising drug for the treatment of epilepsy and related disorders (Orhan et al., 2012). In spite of the benefits offered by this agonist, Retigabine usage was limited in 2017 because it causes undesirable side effects in patients that outweighed its therapeutic prowess (Brodie et al., 2010; Orhan et al., 2012; Brickel et al., 2012). Nonetheless, the use of Retigabine has highlighted the potential of targeting K_V_7 channels in the treatment of hyperexcitability disorders. Furthermore, Retigabine remains to be an important research tool and a model K_V_7 drug.

Two basic premises were considered when defining the deactivation of K_V_7.2/K_V_7.3 channels as the main focus of the present study. Those premises are: 1) K_V_7.2/K_V_7.3 channels activation is typically slower than the duration of a typical neuronal action potential (AP). 2) At the usual resting potential of a neuron, a fraction of these channels has been already opened. The first premise suggests that during a single neuronal AP, the increase in the number of activated K_V_7.2/K_V_7.3 channels is negligible (Corbin-Leftwich et al., 2016). The second premise suggests that the control of excitability by M-currents is carried out by those K_V_7.2/K_V_7.3 channels that are already open in steady state at the resting potential (Corbin-Leftwich et al., 2016). Combined, both ideas imply that the stability of the activated/open channel is critical for the physiological function of K_V_7 channels. Therefore, the present work focuses on studying channel deactivation as a proxy to understand open channel stabilization.

The signaling lipid phosphatidylinositol-4,5-bisphosphate (PI(4,5)P_2_) is essential for the activity of K_V_7 channels. PI(4,5)P_2_ is the most abundant phosphoinositide in the plasma membrane, constituting about 1% of the total lipids in its inner leaflet (Di Paolo and De Camilli, 2006). An increase in the concentration of either PI(4,5)P_2_ or analogous molecules in the intracellular side boosts the open probability of the heteromeric K_V_7.2/K_V_7.3 channel (Li et al., 2005; Telezhkin et al., 2012a). This implies that decreasing the concentration of this phosphoinositide reduces activity by likely reducing the stability of the open channel as shown by single channels recordings of M-currents (Marrion, 1993). Following this idea, a set of experiments were performed in this study to assess the effect of depleting the plasma membrane of PI(4,5)P_2_ on the overall kinetics of deactivation of K_V_7.2/K_V_7.3 channels.

One common strategy to experimentally deplete PI(4,5)P_2_ in cells is to co-express a voltage-sensitive phosphatase (Murata et al., 2005; Okamura et al., 2018). These membrane proteins are phosphoinositide-specific phosphatases with voltage-dependent activity that can efficiently dephosphorylate the inositol head group of these lipids, effectively depleting the membrane of PI(4,5)P_2_ (Villalba-Galea, 2012a; Okamura et al., 2018). In fact, the strong action of VSPs such as Ci-VSP, Dr-VSP, and others can degrade PI(4,5)P_2_ into PI(4)P within a few hundreds of milliseconds, reversibly abolishing K_V_7 currents (Suh and Hille, 2002; Zhang et al., 2003; Villalba-Galea et al., 2009). This ability of shutting down channel activity when employing traditional VSPs would make unfeasible to study the role of PI(4,5)P_2_ on deactivation following prolonged depolarizations because there will be no currents left to study. To circumvent this issue, the chimeric VSP Ci-VS-TPIP (or hVSP1) was used instead of traditional VSPs such as Dr-VSP. Ci-VS-TPIP is a chimeric VSP that contains the voltage-sensor of Ci-VSP and the catalytic domain of the human VSP known as TPIP (Halaszovich et al., 2012) (a.k.a. Hs-VSP1 (Halaszovich et al., 2012; Villalba-Galea, 2012b; Okamura et al., 2018)). It has been already shown that the activation of Ci-VS-TPIP can effectively decrease K_V_7.2/K_V_7.3 channel activity without fully abolishing K^+^-currents (Halaszovich et al., 2012). This property made Ci-VS-TPIP into the ideal VSP for this study.

Retigabine and Flupirtine are able to mitigate the effect of PI(4,5)P_2_ depletion on the activity of K_V_7.2/K_V_7.3 and K_V_7.3 channels (Linley et al., 2012; Kim et al., 2017), while increases in PI(4,5)P_2_ levels reduced Retigabine-induced K_V_7 currents activation (Soldovieri et al., 2016). Although the mechanism underlying this action remains unclear, single channel recordings show that Retigabine increases the lifetime of open state events of K_V_7.2/K_V_7.3 channels, while decreasing the dwelling-time of closed channel events (Tatulian and Brown, 2003). Further, the mean-open-time of the isolated pore of K_V_7.3 channel increases in the presence of Retigabine (Syeda et al., 2015). Furthermore, the deactivation rate of both K_V_7.2/K_V_7.3 and K_V_7.3 channels decreases in the presence of Retigabine (Wickenden et al., 2000; Corbin-Leftwich et al., 2016; Kim et al., 2017). These observations likely indicate that Retigabine increases the stability of the activated/open channel conformation. In addition, Retigabine can also decrease the deactivation rate of channels that are open at typical neuronal resting potential (Corbin-Leftwich et al., 2016). This indicates that Retigabine acts independently of how channels are being opened.

The slowdown of the deactivation rate of the heteromeric K_V_7.2/K_V_7.3 channel can be attributed to conformational rearrangements in the protein that occur following activation. This channel seems to display at least two modes of activity. One mode is observed immediately after activation, and a second mode adopted following prolonged activation (Corbin-Leftwich et al., 2016). Deactivation from this second mode is slower than from the first mode. One intriguing observation is that the effectiveness of Retigabine in decreasing the deactivation rate of K_V_7.2/K_V_7.3 channels is higher as the activation is prolonged (Corbin-Leftwich et al., 2016). This is consistent with the idea that the distinct deactivation rates correspond to distinct conformation of the protein such that the action of Retigabine changes as a function of the deactivation rate. This indicates that the distinct rates of deactivation in K_V_7.2/K_V_7.3 channels can be modes of activity that have distinct kinetic and pharmacological properties.

It has been well established that GqPCR regulation of M-current is mainly mediated by degradation of PI(4,5)P_2_ (Suh and Hille, 2002; Zhang et al., 2003; Suh et al., 2004; Delmas and Brown, 2005; Hernandez et al., 2008a; Hernandez et al., 2009). Activation of GqPCR leads to the hydrolysis of PI(4,5)P_2_ by PLCβ into IP_3_ and DAG. Through this process, the concentration of PI(4,5)P_2_ in the plasma membrane is reduced, decreasing the activity of K_V_7 channels (Suh and Hille, 2002; Zhang et al., 2003; Delmas and Brown, 2005; Suh and Hille, 2005; Suh et al., 2006). The action of PI(4,5)P_2_ seems to promote K_V_7 channel opening by boosting open probability (P_O_) (Delmas and Brown, 2005; Telezhkin et al., 2012a). Consistently, the activity of K_V_7.2-4 channels increases as a function of diC8-PIP_2_ concentration (Delmas and Brown, 2005); diC8-PIP_2_ is a short-aliphatic chain analog of PI(4,5)P_2_. Single channel recordings seemingly show that this increase in P_O_ emerges from both reducing the duration of closed events and increasing the duration of the open events (Delmas and Brown, 2005). On the other hand, decreasing the concentration of PI(4,5)P_2_ decreases that deactivation time constant of K_V_7.2 channels (Chen et al., 2015). These observations suggest that the presence of PI(4,5)P_2_ is critically needed for the stabilization of open channel conformations. Yet, decreasing the mean open event lifetime does not necessarily translate into faster deactivation.

It is important to define the following: Referring to an activated/open channel means channels sojourning between conducting (open) and non-conducting (closed) states, resulting in *open* and *closed* events. In other words, the open probability is higher than zero. While when referring to an open event, it is simply a single channel conducting ions (open event). Considering this, if 1) PI(4,5)P_2_ stabilizes the activated/open K_V_7.2/K_V_7.3 channel and 2) Retigabine is more effective in decreasing the deactivation rate after prolonged activation, it can be expected that depletion of PI(4,5)P_2_ will hamper the stabilizing action of Retigabine on activated/open K_V_7.2/K_V_7.3 channels. To test this hypothesis, the present study analyzed how PI(4,5)P_2_ depletion affected the deactivation of K_V_7.2/K_v_7.3 channels expressed in *Xenopus* oocytes. To decrease the PI(4,5)P_2_ content in these cells, two approaches were employed: One, the co-expression of channels and the chimeric voltage-sensitive phosphatase Ci-VS-TPIP. Two, treatment of oocytes with the phosphoinositide-kinase inhibitor Wortmannin. Here, it is shown that either approach led to increasing the deactivation rate of K_V_7.2/K_V_7.3 channels. Furthermore, it was found that these treatments reduced the effect of Retigabine on the deactivation kinetics. These observations indicated that decreasing PI(4,5)P_2_ concentration had a higher impact on channel deactivation following prolonged activation. These results suggested that K_V_7.2/K_V_7.3 channels in their second mode of activity are more susceptible to PI(4,5)P_2_ depletion than those in the first mode of activity. Extrapolating these results led to postulate that the modal behavior in K_V_7.2/K_V_7.3 channels is likely the physiological target for GqPCR-mediated regulation.

## Materials and Methods

### Preparation of Oocytes and RNA Injections

RNA preparation, *Xenopus laevis* oocyte isolation, preparation, and RNA injection were performed using published methods (Villalba-Galea et al., 2008; Villalba-Galea et al., 2009; Corbin-Leftwich et al., 2016). Animal protocols were approved by the Institutional Animal Care and Use Committees at University of the Pacific and conform to the requirements in the Guide for the Care and Use of Laboratory Animals from the National Academy of Sciences. Ovarian lobules were surgically harvested from frogs purchased from Xenopus 1 (Dexter, MI, U.S.A.). Oocytes were maintained at 16–17°C in a solution of (in mM): 100 NaCl, 1 KCl, 2 CaCl_2_, 1 MgCl_2_ or MgSO_3_, 10 HEPES, 2 Pyruvic acid, pH 7.5, and 20–50 mg/L of gentamycin. Results from many batches of oocytes were combined.

### Electrophysiology

Oocytes were injected with 2 ng of each *in vitro*–transcribed cRNA encoding for the human K_V_7.2 and K_V_7.3 channels and 0.4 ng of the chimeric construct Ci-VS-TPIP. Injected oocytes were incubated at 16–17°C for 2–4 days before recordings. The incubation solution was titrated to pH 7.5 with NaOH and contained (in mM): 99 NaCl, 1 KCl, 10 HEPES, 1 MgCl_2_, 1.8 CaCl_2_, 1 MgCl_2_, 2 Pyruvic acid, and 20–50 mg/L of Gentamycin.

Potassium currents were recorded using the *Xenopus* oocyte Cut-Open Voltage-Clamp (COVC) technique with a CA-1 amplifier (Dagan Corporation, Minneapolis, MN, U.S.A.). The external recording solution contained (in mM): 12 KOH, 88 N-methyl-D-glucamine, 100 Methanesulfonic acid, 10 HEPES, 1 Mg(OH)_2_, and 2 Ca(OH)_2_. The intracellular solution contained (in mM): 100 KOH, 100 Methanesulfonic acid, 10 HEPES, 1 Mg(OH)_2_, and 2 EGTA. Both solutions were titrated to pH 7.4 with Methanesulfonic acid. Retigabine (Alomone Labs, Jerusalem, Israel) was dissolved at 50 mM in DMSO. Wortmannin (Tocris Biosicences, Bristol, U.K.) was dissolved at 20 mM in DMSO. The external solution used for control was added with 0.002%–0.02% (v/v) of DMSO to account for potential effect of the solvent on the activity of the channels. Borosilicate glass electrodes (resistance = 0.2–2.0 MΩ) were filled with a solution containing (in mM) 1,000 KCl, 10 HEPES and 10 EGTA, at pH 7.4 titrated with KOH.

As previously described, voltage control and current acquisition was performed using a USB-6251 multi-function acquisition board (National Instruments, Austin, TX, U.S.A.) controlled by an in-house program coded in LabVIEW (National Instruments, Austin, TX, U.S.A.) (details available upon request). Current signals were filtered at 100 kHz, oversampled at 500 kHz–2 MHz, and stored at 5–25 kHz for offline analysis. Data were analyzed using a custom Java-based software (details available upon request) and Origin 2019 (OriginLab, Northampton, Massachusetts, U.S.A.).

### Exponential Fits and Weighted Average Time Constant

As described in previous studies (Wickenden et al., 2000; Labro et al., 2012; Villalba-Galea, 2014; Corbin-Leftwich et al., 2016), the following two-exponential function was fitted to the deactivating currents:

IDEACT(t)=A1e−tτ1+A2e−tτ2

Where, *A*_1_ and *A*_2_ are the current amplitude associated with each component and τ_1_ and τ_2_ are the corresponding time constants. Fittings were done using Origin 2019 (OriginLab). When needed, the deactivation weighted average time constant (τ_DEACT_) was calculated as

$$\tau_{\mathrm{DEACT}}=\frac{A_{1}\;\tau_{1}+A_{2}\;\tau_{2}}{A_{1}+A_{2}}$$

The fractional amplitude of the second exponential component was calculated as

$$f_{2}=\frac{A_{2}}{A_{1}+A_{2}}$$

T-test were calculated for statistical analysis of the time constants.

It is important to highlight that the two-exponential equation was not derived from a comprehensive kinetic model describing the activity of K_V_7 channels. Instead, it was selected because it can tightly trace deactivating currents. Consequently, the parameters yielded from fitting the equation to such currents can only provide a temporal description of the deactivation process. Meaningful assignment of each individual parameter to any physical process underlying the activity of the channel under study is therefore very limited and even inadequate.

### Molecular Biology

The constructs human KCNQ2 and KCNQ3 in the expression vector pTLN, encoding K_V_7.2 and K_V_7.3 channels, were linearized with MluI and HpaI (New England Biolabs, Ipswich, MA, U.S.A.), respectively. The linearized K_V_7-encoding cDNA was transcribed using a SP6 RNA polymerase kit (Ambion mMessage mMachine, Life Technologies, Carlsbad, CA, U.S.A.). The Ci-VS-TPIP construct in pBSTA was linearized with NotI and transcribed with a T7 RNA ARCA polymerase kit (New England Biolabs). The chimeric phosphatase Ci-VS-TPIP (also known as hVSP1_CiV_) was built by fusing the voltage-sensing domain of the *Ciona instestinalis* voltage-sensitive phosphatase Ci-VSP (Murata et al., 2005) and the catalytic domain of the human voltage-sensitive phosphatase known as TPIP or Hs-VSP1 (Walker et al., 2001). The catalytic selectivity of this construct has been previously characterized, showing that this enzyme decreases PI(4,5)P_2_ in the plasma membrane (Halaszovich et al., 2012).

## Results

### Phosphatase Activity Increases the Deactivation Rate of the K_V_7.2/K_V_7.3 Channel

**Figure 1:**
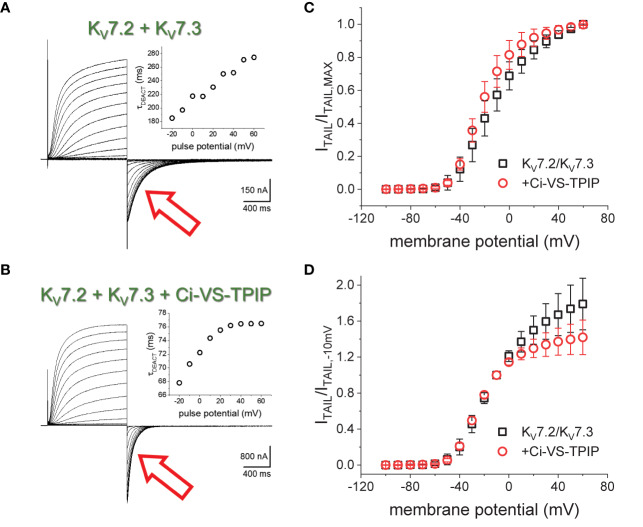
K^+^-currents recorded form *Xenopus* oocytes expressing the heteromeric K_V_7.2/K_V_7.3 channel. **(A)** From a holding potential (H.P.) set at −90 mV, channel activity was probed by applying 1200-ms pulses from −100 mV to +60 mV followed by channels deactivation at −105 mV (red arrow). **(A), inset** The deactivation kinetics was assessed by fitting a two-exponential function to deactivating (“tail”) currents (red arrow), calculating the weighted average time constant as shown in methods (τ_DEACT_), and plotting τ_DEACT_ as a function of the activating-pulse potential amplitude (V_ACT_). Example of a τ_DEACT_-V_ACT_ plot is showed that deactivation speed decreased as V_ACT_ was more positive. **(B)** Co-expressing the chimeric voltage-sensitive phosphatase Ci-VS-TPIP accelerated the deactivation of the K^+^-current (red arrow). (**B, inset**) τ_DEACT_-V_ACT_ plot showed that activation of Ci-VS-TPIP increased the speed of deactivation. **(C)** Voltage dependence of activation was assessed by plotting the amplitude of deactivating current (I_TAIL_) normalized by its maximum value (I_TAIL,MAX_). **(D)** Similar plot than on C. However, I_TAIL_ was normalized by the amplitude of the tail current recorded following an activating pulse to −10 mV (I_TAIL,−10mV_).

K^+^-currents were recorded from *Xenopus laevis* oocytes injected with cRNA encoding the human K_V_7.2 and K_V_7.3 channels. From a holding potential (H.P.) set at −90 mV, K^+^-currents were activated by applying 1200-ms test pulses with amplitudes ranging between −100 mV and +60 mV (**Figure 1**). Subsequently, K^+^-currents deactivated by applying a −105-mV pulses (**Figure 1**, red arrow). A two-exponential function was fitted to the deactivating currents, yielding two time-constants, namely, τ_1_ and τ_2_. Using these values, weighted average deactivation time constants (τ_DEACT_) were calculated and plotted against the amplitudes of the activating pulse potential (**Figure 1A**, insert). As previously reported (Corbin-Leftwich et al., 2016), τ_DEACT_ increased as the amplitude of the activation pulse increased, suggesting that channels became harder-to-close as the activation was stronger. A similar profile was observed when co-expressing the chimeric phosphatase Ci-VS-TPIP (**Figure 1B**). However, τ_DEACT_ were smaller at all activating potentials (**Figure 1B**, insert). These observations indicated that the action of Ci-VS-TPIP increases the rate of deactivation of the heteromeric K_V_7.2/K_V_7.3 channels. This suggested that depleting the membrane of PI(4,5)P_2_ increases the deactivation rate of K_V_7.2/K_V_7.3 channels.

To assess the effect of the phosphatase activity on activation, the normalized deactivating (“tail”) current amplitudes were plotted against the corresponding activating pulse potentials (**Figure 1C**). These I_TAIL_-V_PULSE_ plots showed that the voltage dependence seemed to be altered by the expression of Ci-VS-TPIP. However, the effect at more positive potentials is likely due to the phosphatase own voltage-dependence for its activity; Ci-VS-TPIP activity increases as the membrane potential is made more positive (Halaszovich et al., 2012). Considering this, “tail” current amplitudes were normalized to match the curves at more negative side of the amplitude-voltage relationship at which the phosphatase activity is lower. To do so, tail current amplitudes were normalized with respect to the amplitude observed following a −10-mV activating pulse. The newly generated I_TAIL_-V_PULSE_ plots overlapped at potentials 0 mV and below (**Figure 1D**), suggesting that the action of Ci-VS-TPIP had a low impact in the voltage-dependence for activation of K_V_7.2/K_V_7.3 channels.

### Phosphatase Activity Hampers the Activation-Induced Decrease in the Deactivation Rate

**Figure 2:**
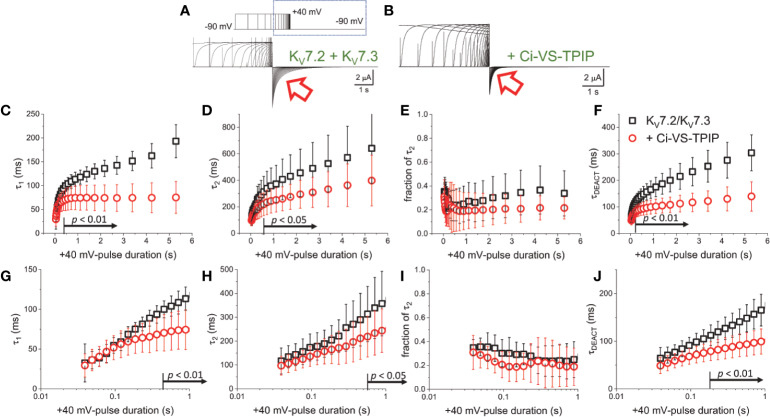
Co-expression of Ci-VS-TPIP accelerates the deactivation kinetic of K_V_7.2/K_V_7.3 channels. **(A)** K^+^-current recorded from *Xenopus* oocytes expressing K_V_7.2/K_V_7.3 channels were activated by applying +40-mV pulses of variable duration from a H.P. set at −90 mV. Deactivation was then driven at −90 mV (red arrow). **(B)** Equivalent recordings were performed from oocytes co-expressing Ci-VS-TPIP. In these recordings, the amplitude of K^+^-currents slowly decayed during activation. This was expected because the activating +40-mV pulse further activates Ci-VS-TPIP, decreasing the PI(4,5)P_2_ concentration in the membrane. **(C, D)** A two-exponential function was fitted to the deactivating currents and the resulting time constants for the first (τ_1_) and second (τ_2_) components of the function were plotted against the duration of the +40-mV activating pulse (t_PULSE_) in the absence (black open squares, *n* = 8) or presence of Ci-VS-TPIP (red open circles, *n* = 13). **(E)** Plot of the relative contribution of the second component of the two-exponential function (fraction of τ_2_) *versus* t_PULSE_ in the absence (black open squares, *n* = 8) or the presence of Ci-VS-TPIP (red open circles, *n* = 13). **(F)** Weighted average time constant (τ_DEACT_) plotted against t_PULSE_ in the absence (black open squares, *n* = 8) or the presence of Ci-VS-TPIP (red open circles, *n* = 13). **(G–J)** Semi-logarithmic version of the plot in **(C–F)**, detailing τ_DEACT_ for t_PULSE_ up to 1 s. the Arrows in panel **(C, D, F–H, J)** indicate the range of t_PULSE_ values at which values were statistically different.

To further assess the effect of Ci-VS-TPIP on the deactivation kinetics of the heteromeric K_V_7.2/K_V_7.3 channel, deactivating K^+^-currents were recorded at −90 mV, following +40-mV pulses of increasing duration (**Figures 2A, B**). As before, a two-exponential function was fitted to the deactivating currents and the yielded time-constants plotted against the duration of the activating pulse (t_PULSE_) (**Figures 2C, D**). For the channels alone, both τ_1_ and τ_2_ increased as t_PULSE_ increased (**Figures 2C, D**, black squares), indicating that channels became harder-to-close as they were activated for longer time. Noteworthy, the increase of both τ_1_ and τ_2_ seemed to ensue in two phases. The first phase occurred within the first 100–200 ms following activation (**Figures 2G, H**, black squares) and the second phase slowly developed thereafter. Likewise, the relative contribution of second exponential component (fraction of τ_2_) decreased during the initial phase, remaining relative constant thereafter (**Figures 2E, J**, black squares). From this analysis, it became evident that the overall tendency of the deactivation kinetics was to slow down as a function of t_PULSE_. An obvious pattern among τ_1_, τ_2_ and the fraction of τ_2_ that could clearly pinpoint any mechanistic underpinnings of this process was not identified. This was not surprising as the two-exponential function used was not derived from a comprehensive kinetic model for activity of this channel. Therefore, to facilitate the interpretation of the data, it was decided to merge these kinetic parameters into a single measure, so generating a τ_DEACT_-*vs*-t_PULSE_ plot (**Figures 2F, J**). These new plots showed the same trend discussed above for τ_1_ and τ_2_ with τ_DEACT_ increasing with t_PULSE_ in two phases. These observations strongly suggest that the open channel became harder-to-close as remained open. These results were consistent with the idea that K_V_7.2/K_V_7.3 channels undergo a transition from the activated/open channel into a more stable conducting configuration.

Next, equivalent recordings were performed from oocytes co-expressing Ci-VS-TPIP (**Figure 2B**). Again, it was observed from the fit of the two-exponential function to the deactivating currents that both τ_1_ and τ_2_ also increased as a function of t_PULSE_ in these oocytes (**Figures 2C, D**, red circles). However, both time constants tended to be smaller than those observed for the channel expressed alone only when the activation was prolonged. These findings indicated that the action of Ci-VS-TPIP has a stronger effect on K^+^-current deactivation when activation is prolonged. Accordingly, the difference between τ_1_ and τ_2_ from oocytes co-expressing the phosphatase and those expressing the channel along were greater as t_PULSE_ was longer, indicating that the effect of PI(4,5)P_2_ depletion was more prominent as activation persisted. In fact, τ_1_ values were significantly smaller only for t_PULSE_ was larger than 454 ms (*p* < 0.01, *d.f.* = 21) (**Figures 2C, G**). Likewise, τ_2_ values were significantly smaller than those in control oocytes for t_PULSE_ longer than 568 ms (*p* < 0.05, *d.f.* = 21) (**Figures 2D, H**).

The trend of the fraction of τ_2_ from oocytes co-expressing Ci-VS-TPIP was not significantly different than that from oocytes expressing the channels alone (**Figures 2E, I**) (*p* > 0.05, *d.f.* = 21). This indicated that the relative contribution of each component remained unaltered by the action of the phosphatase. Given these results, no clear pattern in the three kinetic parameters pointed at any specific mechanism underlying the decrease in the rate of deactivation. Therefore, as before, the kinetic parameters were consolidated into a single one. The calculated τ_DEACT_ from oocytes co-expressing Ci-VS-TPIP were significantly smaller only for t_PULSE_ longer than 186 ms (*p* < 0.01, *d.f.* = 21) (**Figures 2F, J**). This indicated that the effect of Ci-VS-TPIP was more prominent when activation was prolonged, suggesting the stabilization of the open channel was more sensitive to PI(4,5)P_2_ depletion.

### Wortmannin Hinders the Decrease in the Deactivation Rate of the K_V_7.2/K_V_7.3 Channel

One caveat to our interpretation above emerges from the fact that the effect of Ci-VS-TPIP may be due to the voltage- and time-dependence action of this phosphatase. Although the voltage-dependent activity of Ci-VS-TPIP can be observed at voltages as negative as −80 mV (Halaszovich et al., 2012), it is possible that the lack of effect on the deactivation kinetics following short t_PULSE_ is due to low enzymatic activity of this phosphatases, so producing a modest PI(4,5)P_2_ depletion. To address this issue, an alternative approach to decrease PI(4,5)P_2_ in the membrane was employed, treating oocytes with the wide-spectrum phosphoinositide kinase inhibitor Wortmannin. This molecule is a furanosteroid isolated from the fungus *Talaromyces wortmannii* (Nakanishi et al., 1992). Although it is a potent inhibitor of PI-3-kinase, Wortmannin also inhibits phosphoinositide 4-kinase at concentrations at the micromolar range (Nakanishi and Catt, 1995). Because of this, Wortmannin has been shown to effectively decrease the concentration of PI(4,5)P_2_ in the plasma membrane (Suh and Hille, 2002; Zhang et al., 2003; Hirdes et al., 2004; Bernier et al., 2008a; Bernier et al., 2008b; Rodriguez-Menchaca et al., 2012; Dickson et al., 2014; Tang et al., 2015; Kirchner et al., 2017).

**Figure 3:**
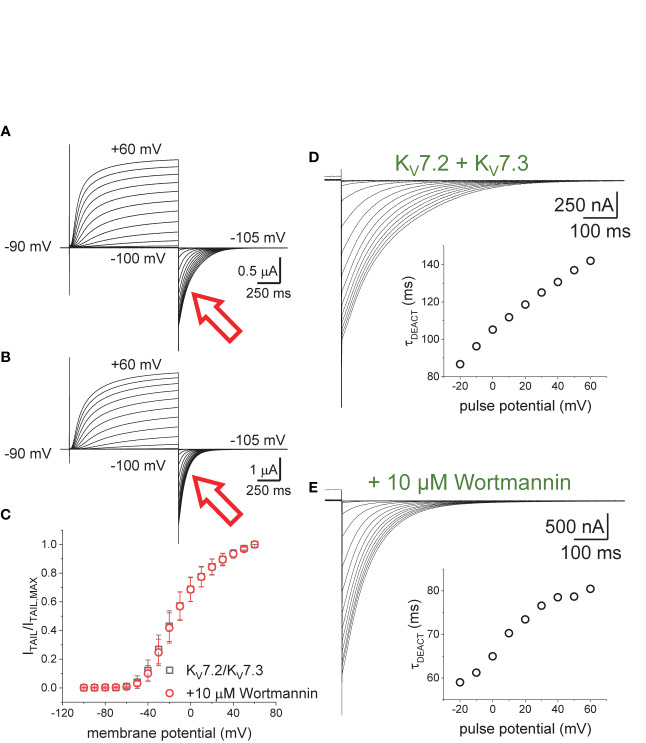
Effect of Wortmannin on the deactivation kinetics of K_V_7.2/K_V_7.3 channels. **(A, B)** *Xenopus* oocytes expressing K_V_7.2/K_V_7.3 channels were incubated for 30–60 min with 10–20 μM of the PI-kinase inhibitor Wortmannin to decrease the concentration of PI(4,5)P_2_. Compared with untreated oocytes (**A**, red arrow), K^+^-current deactivation was faster in oocytes treated with Wortmannin (**B**, red arrow). **(C)** Normalized I_TAIL_-V_PULSE_ shown no difference in voltage-dependence between untreated (*n* = 8) and treated oocytes (*n* = 13). **(D, E)** Examples of deactivating K^+^-currents from oocytes untreated **(D)** and treated **(E)** with Wortmannin. As on **Figure 1**, deactivating K^+^-currents were fitted to a two-exponential function and the calculated τ_DEACT_ values were plotted against pulse potential (V_PULSE_, insets).

Oocytes expressing K_V_7.2/K_V_7.3 channels were incubated in 10–20 µM Wortmannin for 30–60 min; the concentration of Wortmannin varied depending on the sensitivity to the treatment of each oocyte batch. As before, the H.P. was set at −90 mV, K^+^-currents were activated by applying 1,200-ms pulses with amplitudes ranging between −100 mV and +60 mV, and deactivation was driven at −105 mV (**Figures 3A, B**). From these recordings, the amplitude of the deactivating (“tail”) current was plotted against the amplitude of the activation pulse (**Figure 3C**). From these plots it was found that the voltage-dependence for activation of the heteromeric K_V_7.2/K_V_7.3 was unaltered by the treatment with Wortmannin (**Figure 3C**). However, as in the case of co-expressing Ci-VS-TPIP, deactivation of K^+^-currents in treated oocytes (**Figure 3E**) was faster than that of untreated ones (**Figure 3D**). These observations were consistent with the idea that channel deactivation was facilitated by reducing PI(4,5)P_2_ concentration.

**Figure 4:**
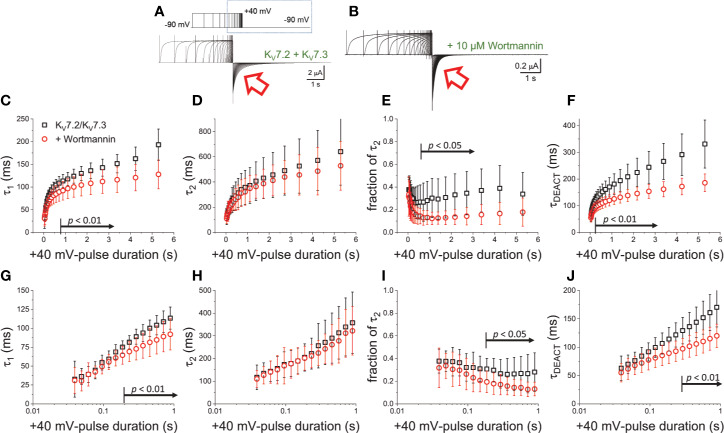
Wortmannin treatment accelerates the deactivation kinetic of K_V_7.2/K_V_7.3 channels **(A)** As on **Figure 2**, K^+^-current recorded from *Xenopus* oocytes expressing K_V_7.2/K_V_7.3 channels were activated with +40 mV pulses of variable duration and then deactivated at −90 mV. **(B)** Equivalent recordings were performed from oocytes treated incubated in 10 µM Wortmannin for 30–60 min. **(C, D)** The deactivating currents (red arrows in A, B) were fitted with a two-exponential function and τ_1_ and τ_2_ were plotted against the duration of the +40-mV activating pulse (t_PULSE_) for untreated (black open squares, *n* = 8) or Wortmannin-treated oocytes (red open circles, *n* = 14). **(E)** Relative contribution of the second component of the two-exponential function (fraction of τ_2_) *versus* t_PULSE_ for untreated (black open squares, *n* = 8) or treated oocytes (red open circles, *n* = 14). **(F)** Weighted average time constant (τ_DEACT_) plotted against t_PULSE_ untreated (black squares, *n* = 8) or treated oocytes (red circles, *n* = 14). **(G–J)** Semi-logarithmic version of the plot in **(C–F)**, detailing τ_DEACT_ for t_PULSE_ up to 1 s. Arrows in panel **(C, E–G, I, J)** indicate the range of t_PULSE_ values at which values were statistically different.

To further evaluate the effect of Wortmannin, K^+^-currents were deactivated at −90 mV following activation by +40-mV pulses of variable duration (**Figures 4A, B**). Fitting a two-exponential function to the deactivating currents yielded τ_1_ values that were significantly smaller than those from untreated oocytes when t_PULSE_ was longer than 200 ms (*p* < 0.01, *d.f.* = 22) (**Figures 4C, G**). In contrast, the values of τ_2_ were not significantly different between untreated and Wortmannin-treated oocytes (**Figures 4D, H**). Yet, the fraction of τ_2_ obtained from recordings with Wortmannin-treated oocytes were significantly smaller with respect to those of untreated oocytes for t_PULSE_ longer than 200 ms (*p* < 0.05, *d.f.* = 22) (**Figures 4E, I**). This suggested that the overall slowdown of the deactivation was increasingly hindered by the action of Wortmannin as a function of t_PULSE_. To test this idea, τ_DEACT_ were calculated and plotted with respect to t_PULSE_ (**Figure 4F**). As before, two phases were found: One initial phase with rapid increase of τ_DEACT_ with respect to t_PULSE_, and a second phase with a slower rate of increase of τ_DEACT_. The calculated τ_DEACT_ increased with t_PULSE_. However, these values were smaller for Wortmannin-treated oocytes with respect to those for untreated oocytes when t_PULSE_ was longer than 291 ms (*p <* 0.01, *d.f.* = 22) (**Figures 4F, J**). Overall, these observations were consistent with the finding that oocytes co-expressing Ci-VS-TPIP, showing that the slowdown of the deactivation kinetics of K_V_7.2/K_V_7.3 channels was impaired by either the action of the phosphatase or by the treatment with Wortmannin. Therefore, it is concluded that PI(4,5)P_2_ depletion has a higher impact on the deactivation rate following prolonged activation.

### PI(4,5)P_2_ Depletion Hampers the Activity-Dependent Stabilization of Open Channels by Retigabine

It is known that depletion of PI(4,5)P_2_ alter the interaction of M-currents with drugs that promote their activity (Zhou et al., 2013). Likewise, such drugs can partially compensate for the decrease in M-currents caused by PI(4,5)P_2_ depletion (Zhou et al., 2013). Retigabine stabilizes the channels’ open states as it increases the dwell time of open events (Tatulian and Brown, 2003; Syeda et al., 2015). Consistently, Retigabine also decreases the deactivation rate of K_V_7.2/K_V_7.3 channels (Main et al., 2000; Linley et al., 2012; Corbin-Leftwich et al., 2016; Yau et al., 2018). Furthermore, at the micromolar range of concentrations (<10 μM), Retigabine is more effective in decreasing the deactivation rate when K_V_7.2/K_V_7.3 channels have been long activated or when opened in steady state at typical neuronal resting potentials (Corbin-Leftwich et al., 2016).

**Figure 5:**
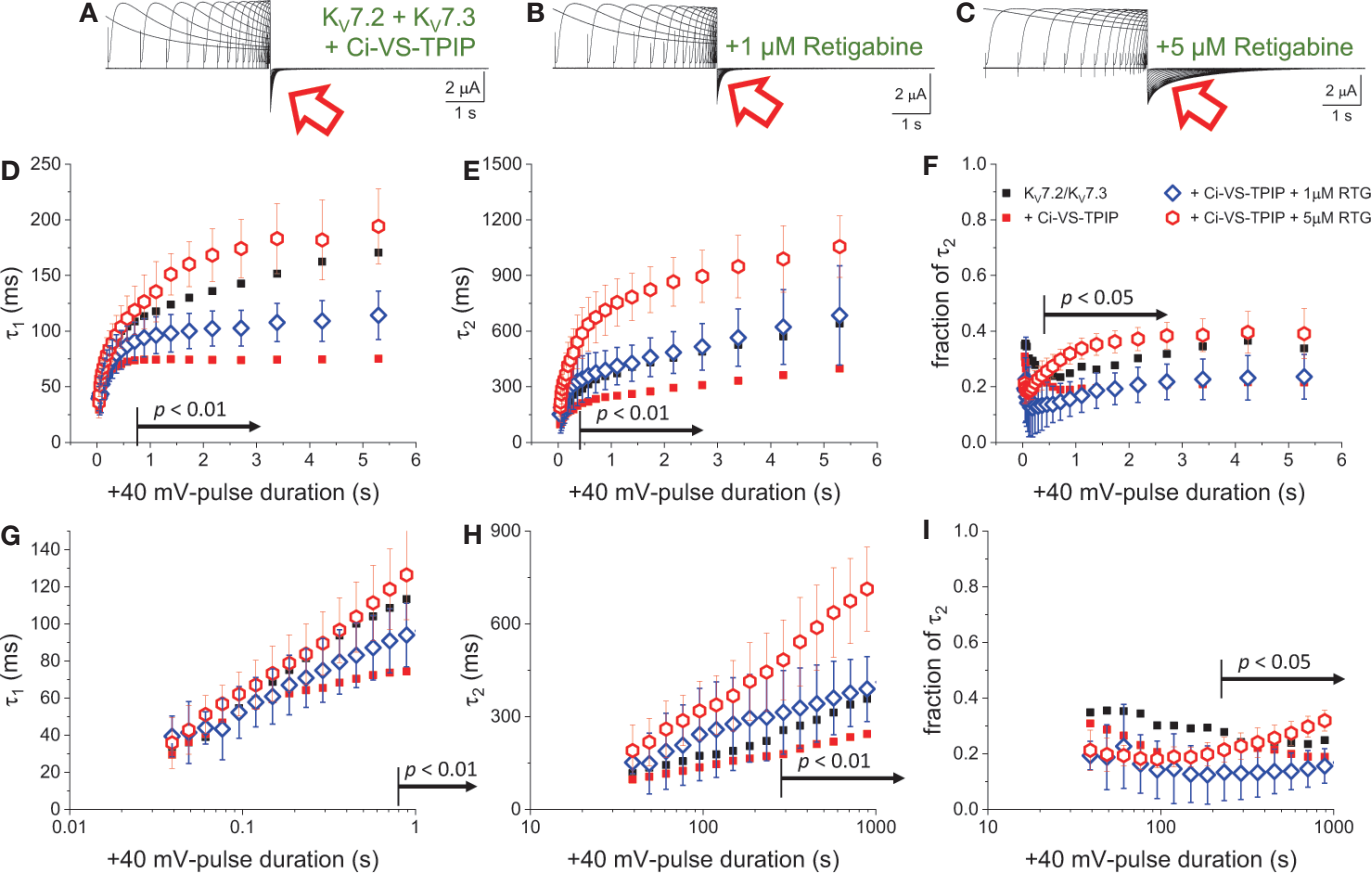
PI(4,5)P_2_ regulates the action of Retigabine. **(A–C)** K^+^-currents were activated with a +40 mV pulses of variable duration in *Xenopus* oocytes expression K_V_7.2/K_V_7.3 channels. Subsequently, the currents were deactivated at −90 mV. These recordings were performed in the absence **(A)** and the presence of 1 µM (blue diamonds, *n* = 6) and 5 µM of Retigabine (red triangles, *n* = 7) **(C)**. **(D–F)** A two-exponential function was fitted to deactivating currents and the yielded τ_1_, τ_2_ and the fractional contribution of the second component (fraction of τ_2_) were plotted against t_PULSE_. (**D–F**, respectively). **(G–I)** Semi-logarithmic version of the plot in **(D–F)**, detailing τ_DEACT_ for t_PULSE_ up to 1 second. For reference, the equivalent values from **Figure 2** were plotted as small black square and small red circles. Black arrows in panels **(D–I)** indicate the range of t_PULSE_ values at which values were statistically different between the recordings in the presence of 1 µM and 5 µM Retigabine.

**Figure 6:**
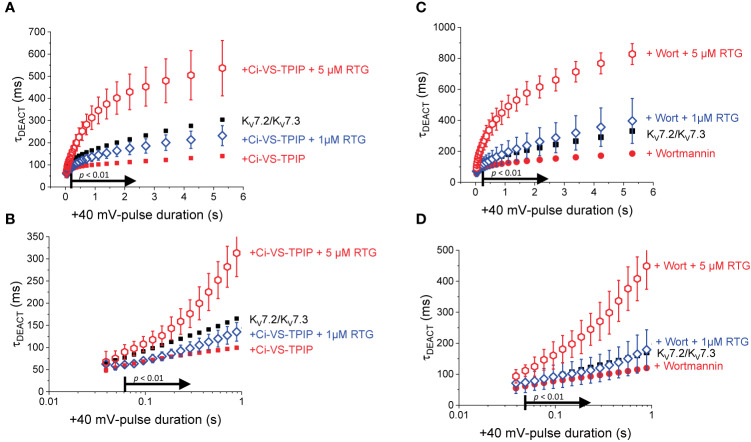
Overall deactivation kinetics as a function of the t_PULSE_. **(A)** As before, to consolidate the analysis of the deactivation kinetics, τ_DEACT_ was plotted against the duration of the +40-mV Pulse (t_PULSE_) for currents recorded in the presence of 1 μM and 5 μM Retigabine (blue open diamonds and red oepn hexagons, respectively). **(B)** The values of τ_DEACT_ were significantly different for t_PULSE_ longer than 61 ms. For reference, the equivalent values from **Figure 2** were plotted as black solid square and red solid circles. **(C, D)** Equivalent τ_DEACT_-t_PULSE_ plots generated from Wortmannin-treated oocytes.

In the presence of 1 μM Retigabine, K_V_7.2/K_V_7.3 channels display up to a two-fold increase in their deactivation time constant when activated at +40 mV for at least 500 ms (Corbin-Leftwich et al., 2016). However, activation with shorter pulses causes negligible changes in the deactivation rate (Corbin-Leftwich et al., 2016). This indicates that the action of Retigabine at low concentrations on deactivation seems to require K_V_7.2/K_V_7.3 channels to be already activated and slowing down their deactivation rate. On the other hand, here it has been found that either co-expression of Ci-VS-TPIP or treatment with Wortmannin impairs the slowdown of the deactivation kinetics. This strongly suggests that PI(4,5)P_2_ depletion would hamper the further slowdown of the deactivation of K_V_7.2/K_V_7.3 channels induced by Retigabine. To test this hypothesis, once again K^+^-currents were recorded from oocytes co-expressing K_V_7.2/K_V_7.3 channels and the chimeric Ci-VS-TPIP (**Figure 5A**). In the presence of 1 µM Retigabine (**Figure 5B**), both τ_1_ and τ_2_ increased (**Figures 5D, E**, blue open diamonds) and were larger than those fitted from recordings in the absence of the drug (**Figures 5D, E**, red solid circles). However, the values of τ_1,_ τ_2_, and the fraction of τ_2_ were all smaller or equal than those observed in oocytes expressing the channels alone (**Figures 5D, E, F**, black solid squares). This indicated that the stabilizing effect of Retigabine was mitigated by the action of the phosphatase. In order to see the slowdown of the deactivation induced by Retigabine, the concentration of the drug was increased to 5 µM (**Figure 5C**). Under this condition, both τ_1_ and τ_2_ increases and were larger than those observed from oocytes expressing channels alone. With respect to oocytes co-expressing the phosphatase and in the presence of 1 µM Retigabine, both τ_1_ and τ_2_ were significantly larger for t_PULSE_ longer than 888 and 363 ms, respectively (both: *p* < 0.01, *d.f.* = 11) (**Figures 5D, E, G, H**, red open hexagons). The fraction of τ_2_ was also significantly higher for 5 µM with respect to 1 µM Retigabine when t_PULSE_ was 232 ms or larger (*p* < 0.05, *d.f.* = 11) (**Figures 5F, I**, red open hexagons). Overall, these observations indicated that PI(4,5)P_2_ depletion impaired the slowdown of the deactivation induced by Retigabine. To summarize these observations, τ_DEACT_-*vs*-t_PULSE_ plots were generated (**Figure 6A**), showing τ_DEACT_ were significantly higher in the presence of 5 µM Retigabine with respect to 1 µM of the drug when t_PULSE_ were larger than 61 ms (*p* < 0.01, *d.f.* = 11) (**Figure 6B**). This reinforce the idea that PI(4,5)P_2_ depletion impairs the action of Retigabine, particularly when channels have been activated for longer periods of time.

To further test the idea that PI(4,5)P_2_ modulates the action of Retigabine, recordings were performed from oocytes expressing K_V_7.2/K_V_7.3 channels alone and treated with Wortmannin. In this case, a similar pattern emerged than with the co-expression of Ci-VS-TPIP. Although the slowdown the deactivation of the K_V_7.2/K_V_7.3 of Wortmannin-treated (**Figures 6C, D**, red solid circles) was boosted by 1 µM Retigabine (**Figures 6C, D**, blue open diamonds), it was not larger than that observed in untreated oocytes (**Figures 6C, D**, black solid squares). Only increasing the concentration of Retigabine to 5 µM produced a significant slowdown in the deactivation kinetics (**Figures 6C, D**, red open hexagons) larger than that in untreated oocytes (**Figures 6C, D**, black solid squares). These combined observations indicated that the action of the Retigabine can be counteracted by either the action of Ci-VS-TPIP or the treatment with Wortmannin. This led to the conclusion that the action of Retigabine on the deactivation kinetics is strongly modulated by PI(4,5)P_2_, particularly following prolonged activation.

## Discussion

Here, it has been shown that either the co-expression of Ci-VS-TPIP or the treatment with Wortmannin had an unambiguous effect on the kinetics of deactivation when it followed prolonged activation. Consistent with previous reports, it seems that K_V_7.2/K_V_7.3 channels undergo a process of stabilization of their activated/open conformation when they are activated for prolonged periods of time. In addition, this underlying process for stabilization seems to require PI(4,5)P_2_. This is consistent with a study showing that K_V_7.2 expressed in CHO cells show a unambiguous decrease in the deactivation rate when co-expressed with the phosphoinositide kinase PI5K, indicating that increasing the concentration of PI(4,5)P_2_ slowdown deactivation (Chen et al., 2015). Yet, it has been shown no change in the deactivation kinetics of M-currents upon application of Muscarine in trypsin-digested isolated bullfrog lumbar sympathetic ganglia (Adams et al., 1982). It is not clear what the reason for this discrepancy is. However, it is to be considered that other factors are in play when studying M-currents in a native system. For instance, in neurons for a sympathetic ganglion, Muscarine activate muscarinic GqPCRs that degrades PI(4,5)P_2_ into DAG and IP_3_, so increasing the concentration of Ca^2+^ through IP_3_ receptors. As Ca^2+^ is known to modulate M-currents, decreasing their deactivation rate (Yu et al., 1994), it is possible that the apparent discrepancy mentioned here arises from a combination of factor playing in the native system.

Another aspect to be considered is the potential effect of PI(4)P when produced by Ci-VS-TPIP after dephosphorylating PI(4,5)P_2_. Although the experiments reported here cannot tell what the effect of PI(4)P would be on the activity of K_V_7.2/K_V_7.3 channels, previous reports have shown that the conversion of PI(4,5)P_2_ into PI(4)P by Ci-VSP and Dr-VSP render the channels inactive (Murata and Okamura, 2007; Villalba-Galea et al., 2009; Kruse et al., 2012). So, in principle, it is unproven, yet reasonable to assume that PI(4)P is ineffective in sustaining the activity of K_V_7.2/K_V_7.3 channels. Consistently, it has been shown that the PI(4)P-analog diC_8_PI(4)P is has a lower potency than the PI(4,5)P_2_-analog diC8PI(4,5)P_2_ to sustain M-current activity (Telezhkin et al., 2012b).

An additional consideration is related to the ability of oocytes to either regenerate or maintain PI(4,5)P_2_ levels. To this, it is important to consider that the current implementation of the COVC technique relies on making a chemical “perforation” in a restricted and small area of the oocyte’s plasma membrane using a mild detergent treatment (Saponin, 0.1% in intracellular recording solution). Thus, the oocytes conserved its integrity. Even when expressing VSPs in oocytes, PI(4,5)P_2_ can be degraded by over and over again as the oocyte regenerates as evidenced by the recovery of the current as VSP deactivation (Villalba-Galea et al., 2009; Halaszovich et al., 2012).

A final consideration on the use of VSPs is the potential effect that they can have on channel activity their enzymatic properties. It has been shown that the inactivated chimeric Ci-VS-TPIP-C363S affects the activation of K_V_7.4 channels expressed in Chinese Hamster Ovary (CHO) cells (Halaszovich et al., 2012). This suggests that it is possible that there is interaction between these proteins. Although this issue was not directly addressed in the present study, the activation of K_V_7 channels in oocytes co-expressing Ci-VS-TPIP did not display any obvious differences with respect to the activation in oocytes expressing the channels alone. Example of that can be seen in **Figure 1**.

### Modal Behavior

The work presented here reinforces the idea that this heteromeric channel displays at least two modes of activity once activated. A first mode that is reached immediately upon activation and a second mode that is reached once activation has been prolonged. Further, the second mode of activity seems to be more stable than the first one because deactivation is slower from it. Furthermore, it was shown that PI(4,5)P_2_ depletion has a relative stronger effect on deactivation as channels are activated for longer periods of time. This suggests that K_V_7.2/K_V_7.3 channels in their second mode of activity are more sensitive to PI(4,5)P_2_ depletion.

Modal gating has been documented in the activity of many types of channels and it has been shown to be critically involved in their function (Nilius, 1988; Marrion, 1993; Naranjo and Brehm, 1993; Zahradnikova and Zahradnik, 1995; Zahradnikova et al., 1999; Magleby, 2004; Tanskanen et al., 2005; Chakrapani et al., 2011; Bicknell and Goodhill, 2016). To understand modal gating, it is essential to define what a “mode” is. In theory, a “mode” constitutes a cluster of interconnected states (typically open and closed) in which a channel dwells for a certain period of time until it transitions to a state belonging to another cluster (Fill et al., 2000). A mode can be seen as “bursts” or “trains” of repetitive opening and closing events. These bursts share similar open probability and lifetimes of open and close channel events. So, a channel can sojourn in one mode and eventually transition into a different mode, changing their open probability and/or events’ lifetime.

One well-documented case of modal gating is the one observed in the activity of the Ryanodine Receptor type 2 (RyR2) (Gyorke and Fill, 1993; Zahradnikova and Zahradnik, 1995; Zahradnikova et al., 1999; Fill et al., 2000). These Ca^2+^-activated channels have at least two modes of open channel activity which are known as the high open probability (HPo) and the low open probability (LPo) modes. Interestingly, RyR2 generally opens into their HPo mode when activated by a rapid increase in Ca^2+^ concentration (Gyorke and Fill, 1993). Then, RyR2 starts reversibly sojourning between modes. When reaching a steady state, HPo and LPo modes are both populated in a Ca^2+^-dependent manner, effectively decreasing the overall open probability (Gyorke and Fill, 1993; Zahradnikova and Zahradnik, 1995; Zahradnikova et al., 1999; Fill et al., 2000). Because the HPo mode is first reached, the activity of RyR2 is always higher immediately after activation with respect to steady state (Zahradnikova et al., 1999; Fill et al., 2000). This is the basis for what is known as “RyR adaptation” which is critical for the control of Ca^2+^-induced Ca^2+^ Release in cardiac myocytes (Gyorke and Fill, 1993; Zahradnikova et al., 1999).

Modal gating is also observed in the activity of the bacterial potassium-selective channel KcsA. This channel displays three modes, namely, High Po, Low Po, and Flickering modes (Chakrapani et al., 2011). Interestingly, mutation of the residue E71 in the selectivity filter of KcsA can strongly modulate the likelihood of observing these modes and their kinetics (Chakrapani et al., 2011). Modal gating is the underlying process of slow inactivation in the activity of KcsA.

For RyR2, KcsA, and some neuronal receptors such acetylcholine and NMDA receptors that undergo desensitization (Nilius, 1988; Magleby, 2004; Bicknell and Goodhill, 2016), modal gating is correlated with the decrease of channel activity as activation is prolonged. However, this is not the case in K_V_7 channels. Modal activity in the heteromeric K_V_7.2/K_V_7.3 channels seems to increase or stabilize their activity as deactivation becomes slower as activation persists.

### Modal Behavior and K_V_7 Pharmacology

The idea of modal gating in the activity of K_V_7 channels was first introduced by N.V. Marrion in 1993, and other subsequently (Marrion, 1993; Selyanko and Brown, 1999). At the single channel level, it has been found that M-currents can display two types of “activity bursts” that where distinguished by their open probability and the lifetime of their open channel events. This observation showed for the first time that K_V_7 channels can operate in more than one mode.

It has been shown from single channel recordings of M-currents that the frequency of longer-living open channel events decreases upon the application of Muscarine (Marrion, 1993). This indicates that modal gating is modulated by PI(4,5)P_2_. Consistently, we observed here that the slowdown of the deactivation is impaired following PI(4,5)P_2_ depletion. This strongly suggest that the modal behavior can be targeted by PI(4,5)P_2_ regulation.

The slowdown of the deactivation is here referred to in terms of “modal behavior” or “modal activity”. These terms have been intentionally used to make a distinction with the concept of “modal gating”. This is because single channel analysis escapes the scope of the present study and is yet-to-be correlated with the change in the deactivation kinetics. Nonetheless, the fact that deactivation becomes slower strongly suggests a modal transition in the activity of these channels as activation is sustained. Supporting the idea of modal activity in K_V_7.2/K_V_7.3 channel is the observation that Retigabine has a stronger impact on deactivation as they are kept activated. Furthermore, this study has shown that PI(4,5)P_2_ depletion also displays a differential effect, having a higher impact on deactivation after prolonged activation. These kinetic, pharmacological, and regulatory types of evidence strongly support the notion of modal activity in K_V_7.2/K_V_7.3 channels.

### PI(4,5)P_2_ and K_V_7 Modal Behavior

The molecular basis for the apparent differential modal sensitivity of the deactivation rate to PI(4,5)P_2_ depletion is yet-to-be defined. However, there are at least two alternatives worth discussing. One is that, since the homomeric K_V_7.2 and K_V_7.3 channels have distinct affinities for PI(4,5)P_2_ (Delmas and Brown, 2005; Telezhkin et al., 2012a), it is possible that the differential modal sensitivity to PI(4,5)P_2_ depletion emerges from the distinct affinities of the subunits. If this was the mechanism, it would imply that the action of PI(4,5)P_2_ in activation and stabilization of the open conformation of the channels would depend on the subunit that this lipid is bound to. An alternative option is based on the existence of multiple binding sites for the PI(4,5)P_2_ within each subunit of the K_V_7.2/K_V_7.3 channel (Zhang et al., 2003; Loussouarn et al., 2003; Hernandez et al., 2008b; Hernandez et al., 2009; Thomas et al., 2011; Telezhkin et al., 2012b; Telezhkin et al., 2013; Chen et al., 2015). In this case, the role of PI(4,5)P_2_ in the processes of activated/open channel stabilization will depend on which binding site is bound to. In this case, the affinity to the binding involved in stabilization would be lower than other sites as stabilization was shown to be more sensitive to PI(4,5)P_2_ depletion. The idea that distinct binding sites have different roles in activation and deactivation is consistent with a previous report suggesting PI(4,5)P_2_ switches position during activation, migrating from the S2-S3 linker to the S4-S5 linker (Chen et al., 2015).

**Figure 7:**
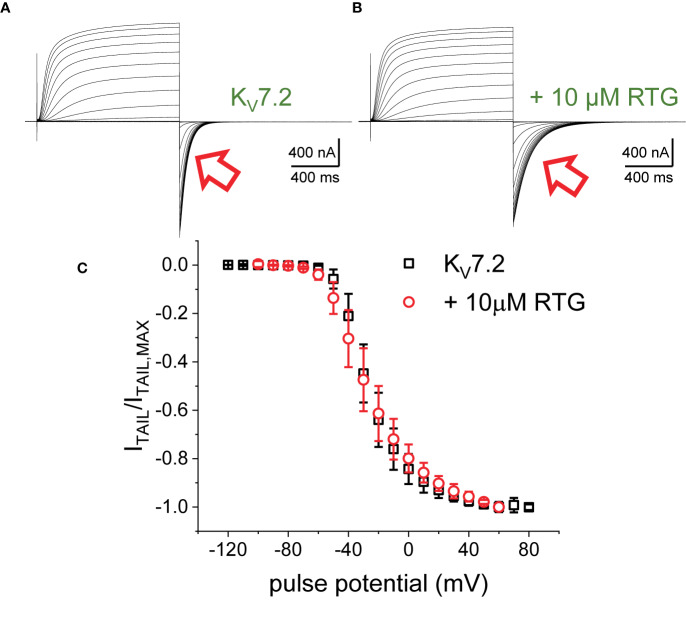
K^+^-currents from oocytes expressing K_V_7.2 channels, before **(A)** and after adding 10 μM RTG **(B)**. The current deactivation slowed down in the presence of the drug (red arrows). **(C)** Deactivation current amplitude (I_TAIL_) were normalized respect the largest current amplitude (I_TAIL,MAX_). The average of the normalized currents were plotted versus the activating voltage (*n* = 15).

**Figure 8:**
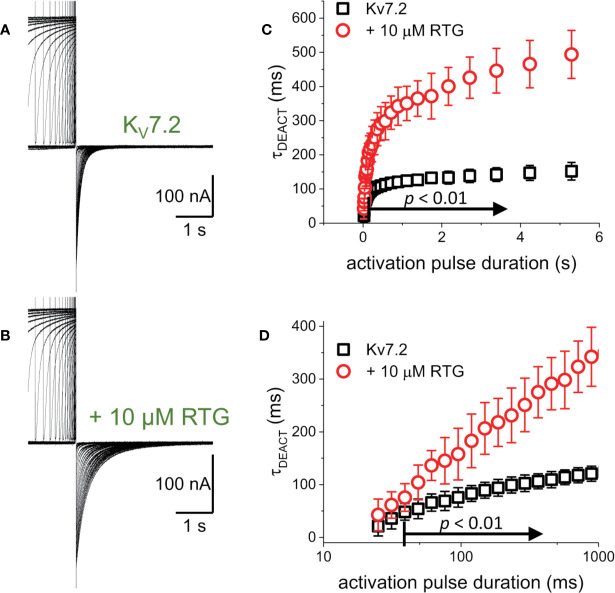
To determine whether the decrease in the rate of deactivation was a unique property of the heteromeric K_V_7.2/K_V_7.3 channel. **(A, B)** K^+^-current recordings from oocytes expressing K_V_7.2 in the absence and presence of 10μM Retigabine (RTG). **(C)** τ_DEACT_ was plotted as a function of t_PULSE_ for the homomeric K_V_7.2. It was found that the slow down of the deactvation kinetics still occurs when K_V_7.2 was expressed alone, suggesting that the hysteretic behavior of these K_V_7 channels was not a property emerging from heteromerization (*n* = 5). **(D)** τ_DEACT_-t_PULSE_ plots in C, replotted with a logarithmic t_PULSE_ to highlight the behavior of τ_DEACT_ at short t_PULSE_ values.

The first putative mechanism implies that inter-subunit differences in affinity are responsible for the increased sensitivity to PI(4,5)P_2_ depletion. The second mechanism implies that multiple sites within each subunit play distinct role in activation and stabilization. Although the approach taken in this study does not allow discriminating between the first inter-subunit and the second intra-subunit multiple-site models, one important distinction between the two models is that the first one would predict that homomeric channels may not display modal behavior. To provide an initial approximation to addressing this issue, K^+^-currents were recorded from *Xenopus* oocytes expressing the homomeric K_V_7.2 channel. As for the heteromeric channel, the H.P. was set at −90 mV, channels activated by 1,200-ms pulses ranging from −100 to +60 mV, and then deactivated at −105 mV (**Figure 7**). As expected, deactivation of the K^+^-currents was slowed down by Retigabine (**Figures 7A, B**, red arrow). At this low concentration of RTG, under the conditions the recordings were made, a negligible shift in the K_V_7.2 channel’s voltage-dependence of activation was observed toward negative potential. Next, the effect of activation on deactivation was evaluated by activating the channels with +40-mV pulse of variable duration, following with deactivation at −90 mV (**Figure 8A**). As before, τ_DEACT_ values were calculated and plotted against the duration of the activating pulse (t_PULSE_) (**Figure 8C**, black open squares). Like for the heteromeric K_V_7.2/K_V_7.3 channel, τ_DEACT_-t_PULSE_ curves exhibited two clear phases in the slowdown of the deactivation of the homomeric K_V_7.2 (**Figure 8C**, black open squares). Addition of 10 µM Retigabine induced an increase in the time constant of deactivation (**Figure 8B**). As before, τ_DEACT_ for short activation pulses (<36 ms) were unaffected by 10 µM Retigabine (*p* < 0.01, *d.f.* = 15) (**Figures 8C, D**, red open circles). These observations led to conclude that the homomeric K_V_7.2 channel also displays at least two modes of activity, implying that modal behavior is intrinsic of each subunit—or at least of the K_V_7.2 subunit—and not arise from it being a heteromeric protein.

### Molecular Basis for Modal Behavior in K_V_7 Channels

Consistent with previous studies (Linley et al., 2012; Kim et al., 2017), results from in the present study show that Retigabine was able to overcome the effect of the partial depletion of PI(4,5)P_2_ in the activity of K_V_7.2/K_V_7.3 channels. In addition to this observation, a novel finding was that both the effect of Retigabine and the effect of PI(4,5)P_2_ depletion are more prominent following prolonged activation. This led to the conclusion that the modal behavior of K_V_7.2/K_V_7.3 channels is modulated by both Retigabine and PI(4,5)P_2_.

Retigabine binds to the channel’s pore, implying that that its effect is likely mediated by this domain. Thus, one emerging question is: what is the mechanism for mode switching? It has been proposed that both the VSD and the pore of channels can display hysteretic behavior (Villalba-Galea, 2017). Hysteresis is a property of physical and chemical systems in which the response of such system to an external force or field depends on the current status of the systems. In general, channels are thought to have a defined voltage-dependence. However, a growing body of evidence shows that voltage dependence is dynamic an dependent on activity itself (Villalba-Galea, 2017). The response to changes in the membrane potential has been shown to be activity-dependent in channels such as *Shaker (*Lacroix et al., 2011*)*, HCN (Mannikko et al., 2005; Elinder et al., 2006; Bruening-Wright et al., 2007; Xiao et al., 2010), K_V_3.1 (Labro et al., 2015), Na_V_ (Bezanilla et al., 1982), and KcsA (Tilegenova et al., 2017), among others. For voltage-gated channels, hysteresis typically manifests as a shift in voltage-dependence toward negative potentials, a decrease in the deactivation rate, or both (Corbin-Leftwich et al., 2016). This implies that activation and deactivation follow distinct pathways or sequence of events. In the case of K_V_7.2/K_V_7.3, deactivation is initially fast, slowing down as the channels remain activated. This indicates that a transition to a more stable open mode of activity occurs as activation persists, implying that deactivation follows a distinct path toward resting states.

A minimal voltage-gated channel is composed of a pore domain and four voltage-sensing domains. So, the question is, which of these domains may cause modal or hysteretic behavior in a channel? Although this question is yet to be answered, there is evidence suggesting that action of both domains can drive these behaviors. For instance, a recent study shows that the bacterial K^+^-channel KcsA can display hysteresis in its activation. This hysteretic behavior is manifested as a shift in their pH dependence for activation (Tilegenova et al., 2017). This means that the affinity for hydrogen ions depends on whether the channel is activated or deactivated (Tilegenova et al., 2017). This study suggests, therefore, that an “isolated” pore can exhibit hysteresis. On the other hand, the voltage-dependence for sensing charge movement of an “isolated” VSD can change as a function of activity, shifting to more negative potential following activation (Villalba-Galea et al., 2008) and/or making deactivation slower (Labro et al., 2012). At this point, it cannot be established if any of these fundamental mechanisms—or any other—is ultimately responsible for modal behavior or hysteresis in the activation of K_V_7.2/K_V_7.3 channels. However, changes in the deactivation rate induced in this study with enzymatic and pharmacological means show a more prominent effect of deactivation, virtually sparing voltage-dependence. This suggests that conformational rearrangements in the pore domain might be a key factor in the modal behavior of K_V_7 channels.

Here, it was shown that there is a link between the regulation by PI(4,5)P_2_ and modal behavior of the K_V_7.2/K_V_7.3 channel. This suggests that GqPCR-mediated modulation may target K_V_7 channel modal behavior. Since the action of Retigabine was also strongly dependent on modal activity, it can be concluded that GqPCR activity is likely an important modulator of K_V_7 channel’s pharmacology.

### Retigabine-Induced Shift in Voltage Dependence

Except for K_V_7.1 channels, it has been commonly reported that retigabine induces a shift in the voltage-dependence for activation of K_V_7 channels, facilitating opening. Under the experimental conditions used in the present study, low concentrations of retigabine (≤10 µM) were able to induce shifts in the voltage dependence for activation of K_V_7.2 and K_V_7.2/K_V_7.3 channels that were smaller than those reported elsewhere. There is not clear explanation for such discrepancy. Yet, there are some considerations that may help explaining it. First, most of the papers reporting the effect of retigabine on K_V_7 channels expressed in oocytes have used the Two-Electrode Voltage-Clamp (TEVC) technique. Employing this technique, we observed in my lab similar shifts in the conductance-*vs*-voltage (G-V) curves of K_V_7.2/K_V_7.3 channels that resembled those reported in the literature. We also observed a large variability in the magnitude of the shift. Furthermore, we noted that the washout of the drug allowed the return of the G-V curve to more positive voltages, but deactivation remained slower. This led us to hypothesize that the variability observed was due to limitation of the TEVC technique (Baumgartner et al., 1999) which was likely exacerbated by the slow deactivation kinetic of the channels. So, it was then decided to change our approach and employ the COVC technique because it offers better space and temporal control of the membrane potential (Taglialatela et al., 1992; Stefani and Bezanilla, 1998). Regretfully, we did not perform further investigation of the issue.

Another important consideration is that the slow kinetic of K_V_7 channels constitutes a challenge in itself when studying slow transition in channel behavior. In this and in our previous study (Corbin-Leftwich et al., 2016), 1 µM of retigabine increased the average deactivation time constant of K_V_7.2/K_V_7.3 channels to approximately 600 ms at −90 mV when following an activating +40-mV pulse of 2 s in duration. This was almost twice the time needed for the channels to close in the absence of the drug. Assuming an exponential deactivation process, a 600-ms time constants means that 3 s was the minimum time required to reach 99% of channel closure. Further, 5 µM of retigabine caused an additional doubling of the deactivation time (Corbin-Leftwich et al., 2016), implying the need of 6 s to allow channels to close. Furthermore, these estimated deactivating times may be even longer if considering that channel closure does not imply full deactivation, as deactivation also involves transition through closed states (Zagotta et al., 1994). Some published studies have used protocols with H.P.s at −80 mV and deactivating potentials at −30/−40 mV. We have observed deactivation time constants of up to 2.5 s at −80 mV in the presence of 1 µM retigabine (Corbin-Leftwich et al., 2016). This indicates that closure could take at least 12 s at such potentials. Being very cognizant of this notion, recordings were performed for the present study providing long periods of time to allow channels to fully deactivate. For instance, 10 s or more for channels to deactivate at −105 mV (**Figures 1**, **3**, and **7**) and at least 16 s when deactivating at −90 mV (**Figures 2**, **4**, **5**, and **8**). It is noteworthy that this is not uncommon feature of channels held active for a long time. For instance, channels like *Shaker*, which is “very fast channels”, can take seconds to fully deactivate (Lacroix et al., 2011). Therefore, we speculate that the source of discrepancy between this and other studies may emerge from differences in the pulse protocols used. Deactivation time constants and periods of recovery are to be carefully designed during electrophysiological studies using voltage-clamp techniques.

### Modeling the Activity of K_V_7.2 Channels

**Figure 9:**
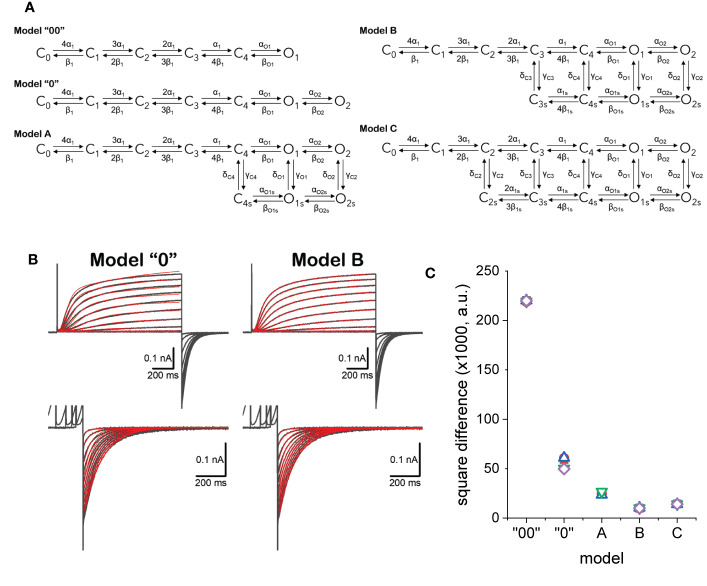
**(A)** Kinetic models considered to describe the activity of K_V_7.2 channels. **(B)** Example of fitted currents data (red traces) overlapping experimentally recorded current traces (black traces). Details in the text. **(C)** Square difference (SD) between the fitted and recorded traces. SD = ∑(I_RECORDED_-I_FITTED_)^2^.

Five models for the activity of K_V_7.2 were evaluated (**Figure 9A**). Models “00” and “0” cannot display modal behavior as they are based on unbranched, sequential kinetic schemes. Models A, B, and C may display modal behavior depending on the rates. Transition in the horizonal direction have rates names α and β which were considered to be functions of the voltage. Transitions in the vertical direction have rates named γ and δ which were considered voltage-independent for simplicity.

Using a framework for analysis previously developed (Villalba-Galea, 2014), each model was simultaneously fitted to activating and deactivating currents recorded from an oocyte expressing K_V_7.2 channels (**Figure 9B**). Activation was driven from a H.P. of −90 mV by voltage pulses with amplitudes ranging from −50 to +40 mV. The deactivation was driven at −90 mV, following +40-mV pulses of increasing duration, ranging from 48 to 4,235 ms. The quality of the fit was assessed by calculating the square difference (SD) between the recording and the fitted traces. For each model, five trials with randomly seeded parameters were used to initialize the fitting. Then, five trials were seeded with the average parameters obtained from the initialization step and let run to obtain the final fitted parameters. The fitted models shown here are part of an ongoing study. In this phase of that study, model B has been the best fitted model according to calculated SD values (**Figure 9C**). This is likely due to the larger number of parameters in model C which requires larger number of iterations.

**Figure 10:**
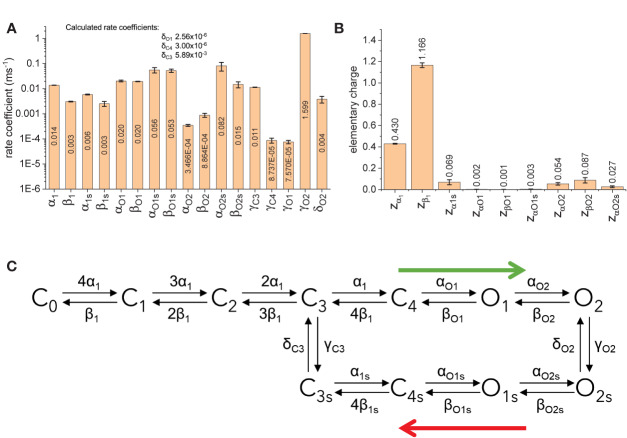
Fitted parameters for model B. **(A)** Rate coefficients for transitions. **(B)** Elementary charges associated to the transtion. **(C)** Simplification of the model B based on the rates. Blue arrows indicate preferred direction immediately after activation (green arrow) and during deactivation following prolonged activation botton (red arrow).

The fitted parameters for model B showed that the rates between states C_0_ through C_4_ are strongly voltage dependent (**Figure 10B**). Accordingly, about 1.6 electron-charges per subunit are associated to these transitions, accounting for 96% of the total sensing charges of the model. On the other hand, transitions between states C_4_, O_1_, and O_2_ display low voltage-dependence, accounting for the remaining 4% of sensing charges. Another important finding is that the transition rates γ_C4_, δ_C4_, γ_O1_, and δ_O1_ were at least one order of magnitude smaller than γ_C3_, δ_C3_, γ_O2_, and δ_O2_ (**Figure 10A**). This made the transitions between C_4_ and C_4s_ and between O_1_ and O_1s_ very unlikely. In addition, given the value of z_α1_ and the transition coefficient α_1_, the rate of transition from C_3_ to C_4_ at +40 mV would be three times larger than γ_C3_ which is the transition rate from C_3_ to C_3s_. Further, α_O1_ was more than three times α_1s_, implying that the transition from C_4_ to O_1_ would readily “drain” the state C_4_, effectively increasing the transition rate from C_3_ to C_4_. Furthermore, γ_O2_ was higher than δ_O2_. This led to conclude that there is a preferred direction in the model, having state O_2_ being populated first following activation and with state O_2s_ being populated as the activation is prolonged. It is important to note that the preferred direction does not violate the micro-reversibility principle. In fact, micro-reversibility was checked at every step of the fit (between 50,000 and 150,000 iterations per trial). From this preliminary study, model B was simplified into an “interpreted” (or effective) model B (**Figure 10C**). The interpreted model B is consistent with the existence of two modes in the activity of K_V_7.2 channels.

### A Final Thought on the Use of a Two-Exponential Function for Quantifying Changes in the Deactivation Kinetics

As in previous studies on the activity of K_V_7 and other K_V_ channels (Wickenden et al., 2000; Labro et al., 2012; Priest et al., 2013; Labro et al., 2015; Corbin-Leftwich et al., 2016), this function was chosen because it is able to tightly trace the deactivation of the K^+^-currents studied here. It is important to stress that the use of a two-exponential function does not mean that the model has two states or modes or ways to be activated or deactivated. This is a misconception that is commonly adopted. Strictly speaking, the number of components in a function describing the behavior of a channel will be equal to the total number of kinetics states (Villalba-Galea, 2014). Thus, if a channel has one closed and two open states, then the number of components should be three. I believe that it is unarguable that channels like those of the K_V_ family have multiple states—say, at least five closed (given that they have four VSDs) and one open (Zagotta et al., 1994). This means that the minimum number of components should be 6, with one of the exponents being zero. When fitting the kinetics of deactivation, this will involve fitting 10 kinetic parameters, namely, time constant and amplitude of each component, plus the baseline (which is the pre-exponential value of the zero-exponent term) (Zagotta et al., 1994; Villalba-Galea, 2014). This argument should suffice to make the point that, beyond the simple temporal description of the deactivation, interpreting the four kinetic parameters yielded by fitting the data to a two-exponential function is inadequate as it contains insufficient information. Furthermore, this is also the reason why assigning physical meaning to these parameters can be misleading and/or inadequate.

## Data Availability Statement

The datasets generated for this study are available on request to the corresponding author.

## Ethics Statement

The animal study was reviewed and approved by IACUC University of the Pacific.

## Author Contributions

The author confirms being the sole contributor of this work and has approved it for publication.

## Conflict of Interest

The author declares that the research was conducted in the absence of any commercial or financial relationships that could be construed as a potential conflict of interest.
